# Recent Advances in the Pharmacological Activities of Dioscin

**DOI:** 10.1155/2019/5763602

**Published:** 2019-08-14

**Authors:** Longfei Yang, Shengnan Ren, Fei Xu, Zhiming Ma, Xin Liu, Lufei Wang

**Affiliations:** ^1^Jilin Provincial Key Laboratory on Molecular and Chemical Genetics, The Second Hospital of Jilin University, Changchun 130041, China; ^2^Department of Surgery, China-Japan Union Hospital of Jilin University, Changchun 130033, China; ^3^Department of Acupuncture and Moxibustion, The Second Hospital of Jilin University, Changchun 130041, China; ^4^Department of Gastrointestinal Nutrition and Hernia Surgery, The Second Hospital of Jilin University, Changchun 130041, China; ^5^Eye Center, The Second Hospital of Jilin University, Changchun 130024, China

## Abstract

Dioscin is a typical saponin with multiple pharmacological activities. The past few years have seen an emerging interest in and growing research on this pleiotropic saponin. Here, we review the emerging pharmacological activities reported recently, with foci on its antitumor, antimicrobial, anti-inflammatory, antioxidative, and tissue-protective properties. The potential use of dioscin in therapies of diverse clinical disorders is also discussed.

## 1. Introduction

**Figure 1:**
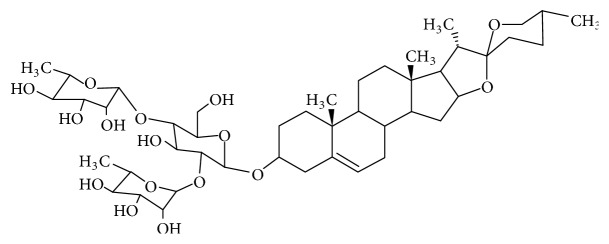
The chemical structure of dioscin. Mw=869.05 g/mol.

Natural products have been an important source of drugs for antifungal, antibacterial, antitumor, and other pharmacological interventions [1, 2]. Recent years have witnessed an increase in the researches on biological activities of compounds from plants. Those from medicinal and edible plant are of special interest, considering their relative safety profiles. One of these compounds is dioscin (Figure 1), the pharmacological activities of which have been renewed a lot in the past decade; therefore, we reviewed here the recent advances of dioscin.

## 2. Sources

As a steroidal saponin, dioscin (diosgenyl 2,4-di-*O*-*α*-L-rhamnopyranosyl-*β*-D-glucopyranoside) could be isolated from various kinds of vegetables and herbs, most of which belong to the family of Dioscoreaceae (Table 1). Some edible plants of* Dioscore* genus (such as* Dioscorea opposita*,* Dioscorea alata,* and* Dioscorea japonica*) serve as starchy staple food in undeveloped countries around the world [3]. The rhizomes of these medicinal herbs are employed to treat atherosclerosis [4], burns [5], fever [5], myocardial ischemia [6], angina pectoris [6], asthma [7], rheumatoid arthritis [7, 8], bronchitis [7], anthrax [8], rheumatic heart disease [8–10], born injuries [9], and gastric disorders [8, 9], to lower cholesterol and triglyceride [11, 12], and to improve circulation [13] in multiple countries.

## 3. Safety Profiles

The dioscin-containing DA-9801 has completed phase II clinical trials for treating diabetic neuropathy in US in 2015 [14], indicating its acceptable safety profiles. Also, dioscin-containing Di-Ao-Xin-Xue-Kang capsules have been employed to treat cardiovascular disease for more than 20 years in China and have been approved for therapeutic use in Netherland since 2012 [15]. Without citing any other toxicity data, the entry for clinical trials and the approval for access into drug market are sufficient to clarify the safety profiles of dioscin for therapeutic use.

As an ingredient of Usukawamarunasu that has been eaten for centuries in Japan, the cytotoxicity of dioscin against HaCaT cells was low, with an IC_50_ of about 100 *μ*M [16]. Dioscin at a concentration of 35 *μ*M did not induce significant apoptosis increase or viability loss in HepG2 2.215 and 293 cells [17]. However, the toxicity of dioscin to many cancer cells was relatively high, with IC_50_ ranging from 2 to 20 *μ*M [18–20]. Although dioscin has shown hepatotoxicity in cultured cells, at the level of *μ*g/mL [21–23], it did demonstrate hepatoprotective effects on liver cells at level of ng/mL [21, 24]. Despite the presence of edema and magnocellular nucleus in hepatocytes of mice given a large dose of dioscin by tail vein injection [25], indicating its potential hepatoxicity, dioscin did exert hepatoprotective effects on mice and rats in many reports, which will be elaborated in the later part [21, 24, 26]. Nonetheless, the toxicity of dioscin must be considered, if administered in large dose. The maximal safe dose of dioscin in rats was reported to be 300 mg/kg/day in a subchronic toxicological assessment [27].

Using pooled human liver microsomes (H161) as the reaction system, dioscin, at a high concentration of 200 *μ*M, showed no inhibitory activities against four UDP-glucuronosyltransferases (UGTs, UGT1A1, UGT1A4, UGT1A9, and UGT2B7) and six of eight Cytochrome P450 (CYP450) enzymes tested (CYP1A2, CYP2A6, CYP2B6, CYP2C8, CYP2C9, and CYP3A4). Even for the two enzymes (CYP2C19 and CYP2D6) inhibited by dioscin, the IC_50_ values were higher than 150 *μ*M [28]. Since CYP 450 and UGTs, besides drug transporters, are associated with drug-drug interactions, these results indicate that dioscin may hardly or very weakly influence the metabolism of other drugs, if used in combination with dioscin, although much more work are needed to be done.

## 4. Anti-Hyperuricemia

As the end product with poor solubility of purine catabolism catalyzed by xanthine oxidase (XO) in human, uric acid in blood is mainly excreted through urine and feces [29–31]. Resulting from overproduction (10%) or aberrantly low excretion (90%) of uric acid through kidney, hyperuricemia is responsible for a majority of gout patients and are associated with diabetes and chronic kidney disease [29, 32, 33]. Thus, besides the classic target XO that plays a critical role in uric acid production, transporters, such as organic anion transporter 1 (OAT1, SLC22A6) and urate transporter 1 (URAT1, SLC22A12) in kidney, might be promising targets for treating hyperuricemia. In rodent models of hyperuricemia induced by potassium oxonate, dioscin could reduce the uric acid level in blood and facilitate the urate clearance, thus ameliorating the renal injuries caused by hyperuricemia [29, 30]. In these studies, dioscin increased the expression of OAT1 and decreased URAT1 and GLUT-9 in kidney, whose function are secretion and re-absorption of urate, respectively [29, 30]. In addition, dioscin has showed weak inhibition on XO activity, which might contributes to its antihyperuricemic effects [30].

And this recently published study identified that it is the metabolite of dioscin, tigogenin, that contributes to the uric acid-lowering effects of dioscin through suppressing URAT1 and promoting ABCG2 (the primary intestinal transporter for uric acid excretion) [29]. Considering that P-glycoprotein transporter (P-gp) is also associated with urate transport in intestine [31] and dioscin has been reported to inhibit P-gp in cancer cells [34–37], dioscin may be a promising candidate for developing antihyperuricemic therapies.

## 5. Antifungal and Antiviral

As an important human fungal pathogen,* Candida albicans* could cause a series of infections that are associated with oral cavity, urogenital tracts, as well as the increasingly used indwelling devices. In some cases,* C. albicans* could cause life-threatening bloodstream infections [38]. The antifungal activity of dioscin against* C. albicans* was first reported by Sautour et al. in 2004 [5, 39] and confirmed later [40], and inhibitory activities against* C. glabrata*,* C. tropicalis* were also reported simultaneously [5, 39, 40]. Later in 2013, the antifungal activity of dioscin against* C. albicans* was attributed to its ability to induce damage to plasma membrane [7]. The cell membrane-disruptive activity of dioscin may be due to the penetration into and accumulation in membrane lipid and the binding to ergosterol, a counterpart of cholesterol in fungal cells [41]. Recently, dioscin was found to be effective against the formation and development of* C. albicans* biofilms, a microbial lifestyle with complex structures and high resistance to antifungal agents [38, 42].

Dioscin could also cause increased membrane permeability and excessive ROS generation in* Saprolegnia parasitica*, a pathogenic fungus in freshwater ecosystem [43]. In addition, dioscin has also been reported to induce abnormal mycelial morphologies of* Pyricularia oryzae*, one famous fungal pathogen of rice [44]. In a word, dioscin has showed significant antifungal activities.

Viruses are important human pathogens that could cause a broad spectrum of diseases. Recently, dioscin was reported to have* in vitro* antiviral activities against adenovirus, hepatitis B virus and vesicular stomatitis virus, although the efficacy varies in different phases of infections [17].

## 6. Antitumor

Dioscin has demonstrated antitumor activities against many kinds of tumors, such as lung cancer, esophageal cancer, gastric cancer, colon cancer, glioblastoma, cervix carcinoma, ovarian cancer, breast cancer, prostate cancer, and leukemia. The target tissues listed can be referred to the review on saponins in 2016 [45]. Recently, the antitumor spectrum of dioscin has been further expanded [46–49]. In most studies, dioscin exerts antitumor effects through intrinsic mitochondrial apoptosis, involving activation of caspase-9 and caspase-3 and reduce in antiapoptotic proteins such as Bcl-2, Bcl-xl, cIAP-1, and Mcl-1 [50–52]. At the same time, the levels of pro-apoptotic proteins (Bak, Bax and Bid) are increased. In the apoptosis induced by dioscin, Ca^2+^ release and increased endogenous reactive oxygen species (ROS) production are common to be found [51, 53–56]. PI3K/Akt/mTOR and p38 MAPK and JNK signaling pathways are also involved in the antitumor activities of dioscin [19, 48, 57, 58]. In pancreatic cancer PANC-1 and ASPC-1 cells, dioscin-induced apoptosis is caused by inhibition of Akt1 signaling, mediated by miR-149-3P, which is one of the 107 microRNAs affected by dioscin [49]. In some cancer cells, extrinsic death receptor pathway was activated by dioscin in addition to mitochondrial pathway [19, 59], although both signaling pathways could converge on downstream pro-apoptotic proteins such as caspase-3, caspase-7 and downstream Bid and Bak [59].

Besides, the apoptosis-inducing effects of dioscin also involves the activation of ERK1/2 and AIF pathway [60, 61], the increase in the levels of NO and inducible NO synthase [20], and demethylation of DAPK1 and RASSF-1*α* gene [47], potentiation of TRAIL-induced apoptosis in human renal cancer cells by downregulation of c-FLIPL [62]. Meanwhile, dioscin was found to inhibit cell proliferation by causing DNA damage and DNA hypodiploidy, and then cell cycle arrest [51, 53, 63]. G_2_/M cell cycle arrest was found in multiple kinds of tumor cells through downregulation of cyclin B1, checkpoint kinase CHK2 and cyclin-dependent kinase CDK1 [19, 20, 63, 64], while S-phase arrest in cells, by way of regulating DNA Topo I, p53, CDK2, and Cyclin A expression [53, 54].

Antitumor effects of dioscin also involves inhibition of migration, invasion and angiogenesis. Dioscin could decrease the CAP-1 related cell migration and invasion, and markedly down-regulate MMP2 and MMP9 expression [53, 58]. DNMT3A, TET2, TET3, ZFPM2 and E-cad were increased while TET1, VIM and MMP9 were decreased by dioscin in breast cancer cells [18]. Angiogenesis is important in the solid tumor growth and migration. Dioscin could suppress the VEGF-induced blood vessel formation and downregulate VEGFR2 and its downstream protein kinases phosphoinositide 3-kinase (PI3K), Src, FAK, AKT, phosphorylated p38 mitogen-activated protein kinase (MAPK), and Erk1/2 expression. Phosphorylated P38 MAPK was upregulated in the human umbilical vein endothelial cells (HUVECs) [65] but decreased in ovarian cancer SKOV3 cells [66], the difference may be related to the different cell types. The* in vivo* studies also confirmed that dioscin could attenuate invasion and migration, decrease tumor size, and extend the lifespan of rats [53, 54].

Dioscin could overcome multidrug resistance and enhance antitumor activity of other drugs. Multi-Drug Resistance (MDR) gene encodes p-glycoprotein (p-170), which is located on the cell membrane and pumps drugs out of cancer cells, resulting in drug resistance. Dioscin was found to significantly inhibit MDR1 mRNA and protein expression and inhibit the NF-*κ*B signaling pathway (via blocking inhibitor *κ*B-*α* (I*κ*B-*α*) degradation), thus strengthening drug absorption in adriamycin- (ADR-) resistant erythroleukemic K562/ADR cells, HepG2/ADR cells [35] and human breast cancer MCF-7/ADR cells, as well as methotrexate- (MTX-) resistant Caco-2 colon cancer cells [34, 36, 37]. Indeed, in rat intestine, the absorption of MTX could be enhanced by dioscin treatment [36]. In lung adenocarcinoma, tyrosine kinase inhibitors (TKI) resistance could be inhibited by dioscin, which acts as dual inhibitor of the MEK/ERK and PI3K/AKT signaling pathways, via suppressing SHP2 expression [67]. Another strategy in melanoma tumors is to utilize the bystander effect of gene therapy, and the main method is introducing the suicide gene into tumor cells and giving drugs. In melanoma cells and* in vivo* samples, dioscin could upregulate the expression of the major components of gap junctions connexins Cx26 and Cx43, resulting in more efficient herpes simplex virus thymidine kinase/ganciclovir- (HSV-tk/GCV-) induced bystander killing [46].

Autophagy also participates in dioscin-induced apoptosis, which could be detected 12 hours after low-dose dioscin exposure and earlier than apoptosis in human lung cancer A549 and H1299 cells and hepatoma Huh7 cells. Dioscin-induced autophagy via ERK1/2 and JNK1/2 pathways. When autophagy was inhibited, dioscin-induced cell apoptosis was significantly enhanced, hence dioscin may provide some benefits for cell survival and act as a cytoprotector [60, 68]. It can also induce autophagy to ameliorate cytotoxicity of ADR by inhibiting PI3K/AKT pathways in MCF-7 and MCF-7/ADR cells [37].

Dioscin also induces differentiation of promyelocytes and melanogenesis in melanoma cells [16, 59]. In AML, dioscin could increase the expression of CCAAT/enhancer-binding protein *α* (C/EBP*α*), which is a critical factor for myeloid differentiation and induce the differentiation of promyelocytes to granulocytes and monocytes [59]. Dioscin was found to reduce expression of tyrosinase, TRP-1, and TRP-2, which could lead to inhibition of intracellular production of melanin, and at the same time, it could decrease the expression of MITF via inhibition of phosphorylation of CREB in the *α*-MSH-induced melanogenesis [16].

Given its significant antitumor activities, dioscin may be a promising candidate for developing anticancer therapies. Moreover, its resistance-sensitizing activity may make dioscin an adjunctive to current anticancer pharmacological therapies.

## 7. Hepatoprotective

As the major organ in human for metabolism (detoxification and assimilation), liver often faces multiple stresses and thus can undergo various pathogenesis in response to those stresses. These pathological changes can manifest as fibrosis, injuries, cholestasis, and fatty liver diseases.

### 7.1. Fibrosis

Hepatic fibrosis, featured by excessive accumulation of extracellular matrix (ECM, mainly collagen and fibronectin), is a long-term process that gradually causes damaged architecture and impaired function of liver, resulting in cirrhosis associated with high mortality and morbidity [106]. Via PI3K/Akt signaling pathway, dioscin can significantly inhibit the synthesis of proteins of ECM, such as Col1a1, Col1a2, Col2a1, Col5a1 and Col6a1 [107]. The degradation of ECM could also be enhanced by dioscin through regulating MMPs and their inhibitors, TIMP [108]. In alcoholic fibrosis, dioscin could mitigate this process through Toll-like receptor 4 (TLR4)/MyD88/NF-kB signaling [109]. Through suppressing p38 MAPK signaling mediated by Sirt1/Nrf2, this saponin could also ameliorate liver fibrosis caused by BDL or DMN [110]. Dioscin could prevent the activation and induce the senescence and apoptosis of hepatic stellate cells, which are critical in liver fibrosis. Besides, the expression of PPAR-*γ*, Nrf2, HO-1, and SOD was increased, while TGF-*β*1/Smad, Wnt/*β* catenin, and MAPK signaling pathways could be suppressed by dioscin to mitigate oxidative stress and alleviate hepatic fibrosis [108, 111], suggesting that dioscin could be a promising candidate with multiple targets in liver fibrosis.

### 7.2. Acute Liver Damage

Dioscin could attenuate the acute liver injury induced by DMN and CCl4 through inhibiting apoptosis, necrosis, oxidative stress and inflammation [112, 113]. The acute liver injury induced by thioacetamide could also be rescued by treatment with dioscin, and this protection involves inhibition on oxidative stress and inflammation through FXR/AMPK signaling pathway [114]. The protective effects of dioscin against liver injury induced by ethanol and acetaminophen are associated with mitochondrial function adjustment, besides apoptosis and inflammation inhibition [115, 116]. Acute inflammatory liver injury induced by LPS could also be attenuated through suppressing TLR4/MyD88 signaling [21]. Virus infections caused by HBV also compromise the function of liver, while the antiviral activity of dioscin implies another kind of protection against damage upon liver [17].

### 7.3. Nonalcoholic Fatty Liver Disease (NAFLD)

Featured by abundant fat storage in liver, NAFLD can progress into cirrhosis and hepatocarcinoma [117]. In NAFLD model of rodents, dioscin could activate the SIRT1/AMPK signaling pathways to regulate genes (such as SREBP-1c and CPT) to attenuate lipid accumulation, suggesting the therapeutic effect of dioscin on NAFLD [24].

### 7.4. Cholestasis

Cholestasis, caused by excessive accumulation of bile salts in liver and featured by primary sclerosing cholangitis, biliary cirrhosis and atresia, could result in increased oxidative stress and apoptosis [26, 118]. In cholestasis models induced by *α*-naphthylisothiocyanate (ANIT), dioscin could clean toxic bile constituents from the liver through upregulation of multidrug resistance-associated protein 2 (Mrp2) and bile salt export pump (Bsep) [26, 118]. Aberrant changes in the expression of other transporters of bile salts (organic anion transporting polypeptides (OATPs), organic cation transporters (OCTs) and Na^+^-taurocholate cotransporting polypeptide (Ntcp)) caused by ANIT could also be prevented by dioscin treatment. Moreover, dioscin could increase the levels of antioxidative powers (such as GSH, GSH-Px, and SOD) and antiapoptotic proteins in animals, as well as in primary cultured cells, to mitigate oxidative stress and to reduce apoptosis in cholestasis [118].

### 7.5. Hepatic Ischemia/Reperfusion (I/R) Injury

Ischemia/reperfusion (I/R) can engender damage to tissues in surgery, transplantation and stroke, while the underlying mechanism involves oxidative stress, inflammation and apoptosis [119, 120]. Although dioscin exerts apoptosis-inducing effects in various kinds of tumor cell lines* in vitro* [19, 48, 51, 54, 61], dioscin did also show protective effects on hepatic I/R injury through suppressing apoptosis in rats [119]. The* in vivo* antiapoptotic activity was exerted through upregulating Bcl-2 and Bcl-x and downregulating Bak, CYP2E1, caspase 3, caspase 9, p53, and PARP [119]. The protection was also associated with decreased ROS, RNS and inflammation [119]. In a word, dioscin has multiple protecting effects on liver. Besides, dioscin can also ameliorate gastric and intestinal I/R injury through inhibiting inflammation and apoptosis, although the signaling pathways affected are different [121–123].

## 8. Lung-Protective

In mouse model of pulmonary fibrosis induced by crystalline silica, dioscin could inhibit TGF-*β*/Smad3 signaling to delay fibrosis [124]. Through regulating immune response, dioscin could reduce the secretion of pro-inflammatory cytokines, which could also be seen in LPS-induced acute lung damage [125]. In addition, dioscin could inhibit the expression and production of MUC5AC mucin by airway epithelial cells, thus contributing to treating inflammatory pulmonary diseases which are associated with mucus hypersecretion [87].

## 9. Nephroprotective

Chronic kidney disease (CKD) severely influences the people's health, with 10-15% of the adults in the world affected, imposing a heavy global burden [126]. CKD also increases the risk for other ailments (such as cardiovascular disease, diabetes, and tumor) and can be triggered by chemicals and food additives including fructose [127, 128]. Renal injury induced by excessive fructose consumption is a well-established rat model for studying the pathogenesis of renal injury [128, 129]. This induction involves decrease in SOD, abnormalities in lipid metabolism and the presence of inflammatory response and fibrosis [128–130]. Fructose-induced renal histopathological changes in rats, such as epithelial cell swelling and vacuolar degeneration of vacuoles and brush border, could be rescued significantly by dioscin treatment [128]. In renal tissue of rat, the inhibitory effects of dioscin against fibrosis, oxidative stress and abnormal lipid metabolism were via restoring the normal expression of Sirt3, the reduced expression of which would result in NF-kB activation and cell death [128, 131]. The expression of TGF-*β*1, which plays an important role in renal fibrosis, could also be inhibited by dioscin via Sirt3 to mitigate renal fibrosis [128].

In inflammatory kidney injury induced by LPS, TLR4/MyD88 pathways was suppressed by dioscin via upregulating miR-let-7i [132]. Moreover, apoptosis and oxidative stress were also inhibited. While in I/R renal injury, TLR4/MyD88 signaling was suppressed by dioscin through upregulating Hsp70 to alleviate damage [133]. Inflammation, as well as oxidative stress, caused by nephrotoxic doxorubicin could be attenuated by dioscin through farnesoid X receptor- (FXR-) mediated signaling [134]. In contrast, cisplatin-induced nephrotoxicity can be suppressed by dioscin through regulating miR-34a/sirtuin 1 (Sirt1) signaling pathways [135].

Since renal damage could be caused directly by hyperuricemia, given the anti-hyperuricemic effect of dioscin, dioscin may offer another kind of nephroprotection [29, 30]. The increased concentration of creatinine in blood (an indicator of renal function) caused by potassium oxonate could be lowered through increased excretion by treatment with dioscin [30]. The pathological lesions caused by potassium oxonate and adenine during hyperuricemia induction in rodent animals could also be mitigated by dioscin [29]. The effects of dioscin on uptake transporters in kidney, such as OATP1B1, OATP1B3, OAT3, and OATP2B1, are different. Dioscin weakly inhibits the activity of OAT3 (major uptake transporter) and OATP1B3 while it stimulates OATP1B1 and OATP2B1, thus facilitating the uptake of steviol glucuronide (natural sweetener used in beverages) mediated by OATP1B1 and OATP2B1 [136]. In addition, through regulating OAT1, URAT1 and OCT2, dioscin shows uricosuric and nephroprotective effects in mice [30].

## 10. Cardioprotective

Clinically, the therapeutic use of doxorubicin for cancer is impeded by its cardiotoxicity, while treatment with dioscin (at the level of ng/mL) could increase the viability of cardiocytes that were exposed to doxorubicin. This kind of protective effects was associated with decreased oxidative insults mediated by downregulation of miR-140-5p in H9c2 cells and rodents [137]. Moreover, apoptosis and oxidative damage in H9c2 cells induced by I/R could also be suppressed by dioscin, despite that dioscin could induce apoptosis in various cancer cells [138].* In vivo* protective effects of dioscin against myocardial I/R injury in rats were exerted through upregulation of connexin 43 [139]. In addition, dioscin could also suppress cardiac hypertrophy induced by angiotensin II infusion through downregulating MAPK and Akt/GSK3*β*/mTOR pathways, improving the impaired cardiac function [140].

## 11. Cerebral Protection

During treatment of ischemic stroke, which causes a large proportion of morbidity and mortality worldwide, reperfusion can cause damage to brain [141]. Dioscin could attenuate cerebral I/R injury through inhibiting TLR4/MyD88/TRAF6 signaling mediated by HGMB-1 suppression [141]. In this process, cerebral inflammation can also be inhibited by dioscin [141, 142]. Combined with baicalein, dioscin could mitigate the malfunction of spatial learning memory caused by cerebral I/R in mice [143]. This combination could also contribute to hippocampal plasticity through multiple signaling pathways, thus facilitating post-ischemic rehabilitation associated with stroke [144]. One of the latest researches also showed that dioscin could protect hippocampus from endotoxemia induced neuroinflammation [145].

## 12. Antiatherosclerosis

Adhesion molecules-mediated interaction between monocytes and endothelial cells plays an important role in the initial phase of atherosclerosis, which is an inflammation-associated vascular disease [146]. Dioscin could suppress the expression of adhesion molecules (such as VCAM-1 and ICAM-1) and the synthesis of endothelial lipase in HUVECs stimulated by inflammatory cytokine TNF-*α*, through inhibiting NF-kB nuclear translocation and activation [146].

Meanwhile, the secretory inhibition of IL-1*β*, IL-6, and TNF-*α* via modulation of NK-kB can attenuate the inflammatory response in atherosclerotic plaque [147]. Another consequence of NF-kB inhibition is that LOX-1 expression would be inhibited by blocking the binding of LOX-1 promoter with NF-kB [147]. The formation of macrophage-derived foam cells, which constitute the lipid core and is a characteristic of early atherosclerosis, could also be inhibited by dioscin treatment [147]. In rat model of atherosclerosis induced by high-fat diet, dioscin could decrease the lipid level in blood and aortic vessels [147]. Dioscin has also been used as a positive control for its lipid-lowering effect [77]. This makes dioscin a promising molecule of anti-atherosclerotic and lipid-lowering property.

## 13. Anti-Inflammatory

Inflammation, as the host response to detrimental stimuli, are mediated by pro-inflammatory cytokines such as TNF-*α*, IL-1*β* and IL-6, which are secreted by immune cells recruited to injury sites. Sustained inflammatory cytokines release and reaction result in chronic inflammation, which is featured by excessive immune cell infiltration and is the major cause of many diseases [148].

TNF-*α*, IL-1*β*, and IL-6 secretion in HUVECs, macrophages, NRK-52E and HK-2 cells, can be inhibited by dioscin [135, 146, 147]. The mRNA levels of TNF-*α*, IL-1*β* and IL-6 can also be suppressed in mice and rat model of multiple diseases, such as acute liver injury [112], liver fibrosis [108], obesity [149], cerebral and intestinal I/R injury [121, 141], and inflammatory injuries of kidney, liver, and lung [21, 125, 132], thus attenuating inflammatory damage.

Acute neuroinflammation caused by endotoxemia could be mitigated by dioscin treatment, which is associated with improved 5-HT metabolism [145]. Protecting roles of dioscin against systemic inflammatory response syndrome has also been demonstrated, though inhibiting TLR2/MyD88/NF-kB signaling pathway in mice and rats [150].

In adipocytes, dioscin could also increase the expression of adiponectin, a hormone known to own anti-inflammatory effects, thus possibly exerting another kind of indirect anti-inflammatory effect [151].

## 14. Antiarthritic

As a chronic inflammatory disease caused by disorder of individual immune system and featured by overgrowth of synoviocytes and fibroblast-like synoviocytes, rheumatoid arthritis may engender articular degeneration and damage, affecting heavily public health given its prevalence [152, 153]. The* in vitro* inhibitory effects of dioscin on synovial cell hyperplasia induced by IL-1*β* has been reported [154]. In collagen induced arthritis in mice, dioscin could also palliate the synovial hyperplasia and mitigate the inflammatory response, through increasing the expression levels of p-STAT6 and GATA3 and decreasing p-STAT4 in synovium, which is mediated by regulating the balance between Th1 and Th2 cells [152].

In gouty arthritis induced by monosodium urate crystal, the* in vivo* therapeutic effects of dioscin may be accomplished by inhibiting the increase in stromal cell-derived factor-1 (SDF-1), CXCR4 (receptor of SDF-1) and p38 MAPK signaling in synovial tissue of rats [154]. Considering that SDF-1/CXCR4 signaling is involved in the pathogenesis of rheumatoid arthritis (synovium-derived SDF-1 was increased over 10-fold in RA patients), dioscin may exert multiple protective effects on arthritis [152, 154, 155].

Osteoarthritis, emanating from damage to cartilage and underlying bones, can cause pain, stiffness, and deformity and affect millions of people all around the world [156, 157]. In monosodium iodoacetate induced osteoarthritis, the degradation of ECM by MMP13, CHOP-mediated apoptosis of chondrocytes, inflammation, ER stress and oxidative stress could be inhibited by dioscin. Wnt/*β*-catenin signaling, which activates catabolism in chondrocytes, could also be downregulated by dioscin, while PPAR*γ*, whose activation can lower the production of catabolic factors and ameliorate the oxidative stress, could be upregulated, thus contributing to the protecting effects of dioscin against osteoarthritis [156].

## 15. Antiobesity and Diabetes

Obesity is often associated with increased risk for a series of diseases including type 2 diabetes mellitus (T2DM), certain cancers, osteoarthritis, cardiovascular, and respiratory diseases. Worldwide, the incidence of obesity is growing while the drug is limited, highlighting the necessity for developing new drugs [158].

Dioscin inhibits porcine pancreatic lipase* in vitro* with an IC_50_ of 20 *μ*g/ml (thus inhibiting the absorption of fat) while* Dioscorea nipponica* Makino powders containing dioscin inhibits the rise of blood triacylglycerol concentration induced by oral gavage of corn oil emulsion in mice [158]. In rats, this powder also lowers the increase in body weight and adipose tissue caused by high-fat diet (HFD), through enhanced excretion of fat through feces other than decreased food intake [158]. This effect was also confirmed by the later researches, which reported that HFD-induced obesity, as well as NAFLD, could be suppressed by dioscin [149, 151]. In the later research, the anti-obesity effect of dioscin was due to the elevated energy expenditure rather than the increased physical activity in obese and HFD mice [149].

At concentrations without obvious cytotoxicity, dioscin can inhibit insulin exposure-initiated differentiation by delaying cell cycle progression and suppressing the phosphorylation of ERK1/2 and p38 rather than c-JNK in differentiating preadipocytes. Dioscin can also inhibit adipogenesis through inducing phosphorylation of AMPK, which stimulates the catabolic process of fatty acid to facilitate the energy production [151].

Besides its anti-obesity effects, dioscin might exert beneficial effects on diabetes, which represents a heavy burden on public health system. In a study published in 2018, dioscin has been shown to promote *β* cell proliferation through Wnt/*β*-catenin pathway and attenuate the impairments (decreased viability and increased apoptosis) of *β* cells induced by high-glucose treatment [159]. Moreover, in HFD-fed mice, the insulin resistance in adipose tissues could be mitigated by treatment with dioscin through insulin receptor substrate 1 (IRS-1)/PI3K/Akt pathway [160]. Taken together, dioscin may has potential for treating obesity and diabetes, although it has a very long way to go.

## 16. Antioxidative Stress

Oxidative stress plays a considerable role in the pathogenesis of a variety of diseases and injuries such as pulmonary fibrosis [161], CKD [128], inflammatory lung injury [125], and cisplatin- and doxorubicin-induced kidney injury [134, 135].

SOD2, which contributes to removing superoxide during oxidative stress [162], could be activated by Sirt3, which could be upregulated by dioscin in renal tissue [128]. The inducing effects of TGF-*β*1 on ROS production and myofibroblast differentiation could also be suppressed by overexpression of Sirt3 [163]. In cardiac myoblast H9c2 cells, dioscin treatment could attenuate oxidative stress through increasing SOD expression [138]. Dioscin could also increase the* in vivo* expression of other antioxidant enzymes such as HO-1, Nrf2, and GSH-Px in rats and lower the levels of MDA, NO and iNOS [108, 119, 125, 149]. In a word, the inhibitory effects of dioscin on oxidative stress can also be seen in a variety of disease models, such as hepatic fibrosis, I/R injuries, cardiac hypertrophy, and drugs-induced toxicity [110, 112, 119, 137, 140, 141].

## 17. Antiosteoporosis

Osteoporosis, featured with loss of bone tissue that associated with raised risk of fracture, is caused by imbalanced bone homeostasis towards bone resorption by osteoclasts [164]. This imbalance could be restored by induction of osteoblast activity and suppression of osteoclast function [164, 165].

Dioscin could facilitate osteoblastic proliferation and differentiation through low-density lipoprotein receptor related protein 5 (Lrp5) and estrogen receptor (ER) pathways in mouse pre-osteoblast-like MC3T3-E1 cells and in human osteoblast-like MG-63 cells [164]. Apoptosis in osteoblasts could also be suppressed by dioscin through increasing the expression of the anti-apoptotic Bcl-2, thus promoting the bone mass maintenance [164]. Another beneficial effect of dioscin on osteoporosis is decreasing the expression of RANKL (receptor activator of NF-kB ligand, secreted by osteoblast and stromal cells) which induces NF-kB signaling pathway through binding to and activating osteoclast surface receptor RANK and subsequent Akt phosphorylation [164–166]. Phosphorylated Akt stimulates NF-kB and NFATc1 activity, which are critical for osteoclastic survival and differentiation. Therefore, dioscin could also inhibit bone resorption by osteoclasts though down-regulating the Akt signaling cascades [165]. The protecting effect was also confirmed in bone destruction mice model induced by LPS [165]. The TRAF6 (tumor necrosis factor receptor-associated factor 6) signaling, which initiates pathways associated with osteoclast activation (such as MAPKs and Akt) and can be activated by LPS/TLR4, could also be inhibited by dioscin [166]. The decrease in osteoprotegerin (OPG, the decoy receptor for RANKL) mRNA level and OPG/RANKL ratio in femurs induced by ovariectomy could be rescued by dioscin in rodents, albeit the effects on reversing osteoclastogenesis were in vain [166].

## 18. Other Bioactivities

Dioscin could synergize with oxyresveratrol in suppressing tyrosinase (the enzyme critical in melanogenesis), using L-tyrosine and L-DOPA as substrates* in vitro*, suggesting its potential use as cosmetic whitening agent and for preventing melanin hyperpigmentation [95]. Later, in B16 murine melanoma cells, dioscin was shown to inhibit the melanin production induced by *α*-melanocyte-stimulating hormone (MSH), and this inhibition was due to the decreased expression of tyrosinase, tyrosinase-related protein-1 (TRP-1) and TRP-2 via downregulation of phosphor-CREB and microphthalmia-related transcription factor (MITF) [16].* In vitro* inducing effect of dioscin on growth hormone release in rat pituitary cells has been reported to be strong, with an approximately 17-fold stimulation of release [104]. Dioscin has also showed anti-depressant effects through increasing 5-hydroxytryptamine (5-HT) in hippocampus in mice [145].

Dioscin also showed strong anthelmintic activity against the aquacultural ectoparasite* Dactylogyrus intermedius*, with an EC_50_ of 0.44 mg/L, which is more effective than the positive used [167].

## 19. Conclusions

Recent years have seen a lot of reports on the beneficial effects of dioscin, including antitumor, hepatoprotective, antiviral, nephroprotective, cardioprotective, antifungal, anti-hyperuricemia activities. Although dioscin falls out of the rule of five due to its large molecular weight, it does fall into the sweet scope where the balance between protein-protein interaction (PPI) inhibition and oral bioavailability may be found [168]. Moreover, many derivatives with diverse modifications have been synthesized, leading to great ease to explore the structure-activity relationship for optimization [169–171]. Considering the safety profiles shown by this compound, dioscin could be a promising candidate for developing therapies against multiple disorders.

## Figures and Tables

**Table 1:** Occurrence of dioscin in plants.

Family	Species	Plant parts	References
Dioscoreaceae	*Dioscorea nipponica*	Rhizomes	[7, 69]
Dioscoreaceae	*Dioscorea panthaica*	Rhizomes	[6, 8]
Dioscoreaceae	*Dioscorea spongiosa*	Rhizomes	[29]
Dioscoreaceae	*Dioscorea alata*	Rhizomes	[70]
Dioscoreaceae	*Dioscorea bulbifera*	Tubers	[17]
Dioscoreaceae	*Dioscorea cayenensis*	Rhizomes	[5, 71, 72]
Dioscoreaceae	*Dioscorea zingiberensis*	Rhizomes	[73, 74]
Dioscoreaceae	*Dioscoreae septemlobae*	Rhizomes	[30]
Dioscoreaceae	*Dioscorea villosa*	Roots	[18]
Dioscoreaceae	*Dioscorea mangenotiana*	Tubers	[71]
Dioscoreaceae	*Dioscorea rotundata*	Tubers	[71]
Dioscoreaceae	*Dioscorea villosa*	Roots	[75, 76]
Dioscoreaceae	*Dioscorea pseudojaponica*	Tubers	[77, 78]
Dioscoreaceae	*Dioscorea batatas*	Rhizomes	[79, 80]
Dioscoreaceae	*Dioscorea parviflora*	Rhizomes	[81]
Dioscoreaceae	*Dioscorea futschauensis*	Rhizomes	[82]
Dioscoreaceae	*Dioscorea collettii*	Rhizomes	[43, 44]
Dioscoreaceae	*Dioscorea tokoro *Makino	Rhizomes	[83]
Dioscoreaceae	*Tamus communis*	Rhizomes	[84, 85]
Liliaceae	*Allium ampeloprasum*	Bulbs	[86]
Liliaceae	*Asparagus cochinchinensis*	Roots	[87]
Liliaceae	*Ophiopogon japonicus*	Flowers	[88]
Liliaceae	*Paris Chinensis*	Unspecified	[89]
Liliaceae	*Paris polyphylla*	Rhizomes	[90, 91]
Liliaceae	*Polygonatum sibiricum*	Rhizomes	[19]
Liliaceae	*Polygonatum Zanlanscianense *Pamp	Roots	[50, 92, 93]
Liliaceae	*Smilax bockii *Warb	Roots	[94]
Liliaceae	*Smilax china*	Rhizomes	[95]
Liliaceae	*Smilax excelsa*	Rhizomes	[96, 97]
Liliaceae	*Smilax menispermoidea*	Rhizomes	[98]
Liliaceae	*Trillium Tschonoskii Maxim*	Rhizomes	[99]
Solanaceae	*Solanum heteracanthum*	Roots	[100]
Solanaceae	*Solanum incanum*	Roots	[100]
Solanaceae	*Solanum indicum*	Whole plant	[101]
Solanaceae	*Solanum melongena L. Usukawamarunasu*	Fruits	[16]
Solanaceae	*Solanum rostratum*	Aerial parts	[102]
Arecaceae	*Borassus flabellifer*	Male flowers	[103]
Convallariaceae	*Smilacina atropurpurea*	Rhizomes	[40]
Fabaceae	*Trigonella foenum-graecum*	Seeds	[104]
Zygophyllaceae	*Tribulus terrestris*	Arial parts	[96, 105]
